# Genome-Wide Analysis of *Oceanimonas* sp. GK1 Isolated from Gavkhouni Wetland (Iran) Demonstrates Presence of Genes for Virulence and Pathogenicity 

**DOI:** 10.22074/cellj.2015.6

**Published:** 2015-10-07

**Authors:** Laleh Parsa Yeganeh, Reza Azarbaijani, Hossein Mousavi, Seyed Abolhassan Shahzadeh Fazeli, Mohammad Ali Amoozgar, Ghasem Hosseini Salekdeh

**Affiliations:** 1 Molecular Bank, Iranian Biological Resource Center (IBRC), ACECR, Tehran, Iran; 2 Faculty of Basic Sciences and Advanced Technologies in Biology, University of Science and Culture, Tehran, Iran; 3 Microorganism Bank, Iranian Biological Resource Center (IBRC), ACECR, Tehran, Iran; 4 Agricultural Biotechnology Research Institute of Iran, Karaj, Iran; 5 Department of Molecular Systems Biology, Cell Science Research Center, Royan Institute for Stem Cell Biology and Technology, ACECR, Tehran, Iran

**Keywords:** Pathogenicity, Virulence Factors, Halotolerant

## Abstract

**Objective:**

The bacterium *Oceanimonas* sp. (*O.* sp.) GK1 is a member of the *Aeromonadaceae* family and its genome represents several virulence genes involved in fish and human pathogenicity. In this original research study we aimed to identify and characterize the putative virulence factors and pathogenicity of this halotolerant marine bacterium using genome wide analysis.

**Materials and Methods:**

The genome data of *O.* sp. GK1 was obtained from NCBI. Comparative genomic study was done using MetaCyc database.

**Results:**

Whole genome data analysis of the *O.* sp. GK1 revealed that the bacterium possesses some important virulence genes (e.g. ZOT, RTX toxin, thermostable hemolysin, lateral flagella and type IV pili) which have been implicated in adhesion and biofilm formation and infection in some other pathogenic bacteria.

**Conclusion:**

This is the first report of the putative pathogenicity of *O.* sp.GK1. The genome wide analysis of the bacterium demonstrates the presence of virulence genes causing infectious diseases in many warmand cold-blooded animals.

## Introduction

*Oceanimonas* sp. (*O.* sp.) GK1 (IBRC-M10197) was noticed previously for its high capacity of poly-ß-hydroxybutyrate (PHB) production under extreme growth conditions (1). The bacterium belongs to the *Aeromonadaceae* family which comprises of five genera: *Aeromonas*, *Oceanimonas*, *Oceanisphaera, Tolumonas and Zobellella* (2) and contains several important human and animal pathogens. The pathogens belong mostly to the *Aeromonas* genus and cause different kinds of diseases in many warmand cold-blooded animals (3, 4). Travelers’ diarrhea, cellulitis or wound infections due to traumatic injury in aqueous environment, septicemia and various other infections such as urinary tract infections, surgical wound infections, meningitis, peritonitis and endocarditis are diseases that are caused by *Aeromonas* species (5-8).

Virulence factors, known as one of the important components of pathogenic bacteria, are produced and delivered due to hostpathogen interactions and evoke host cell immune response (9). Because of the key role of virulence factors in pathogenesis, a vast number of investigations have been carried out globally to identify the main virulence factors and their function in bacterial infectious diseases. Virulence factors in bacteria may be encoded on chromosomal DNA, bacteriophage DNA, plasmids or transposons in either plasmids or the bacterial chromosome (10). Adhesins (11- 14), endotoxins (15, 16), exotoxins (17-19), enzymes (20-23), modulins (24) and capsules (25) are some types of virulence factors.

Deciphering the *O.* sp. GK1 genome was the first attempt to discover the unique genomic capabilities and important features of the *Oceanimonas* genus (1). Analysis of the genome revealed some medically as well as environmentally important features of the bacterium which have not been considered and reported before. So far, there has been no report or evidence for pathogenicity of the members of *Oceanimonas*.

The present study is the first comprehensive attempt to characterize the pathogenic capabilities of O. sp. GK1 via *in silico* analysis. In order to investigate the potential of the bacterium for pathogenesis, comparative genomic study was performed with three genomes of closest pathogenic *Aeromonas* species. Primary annotation and *in silico* analysis of the genome revealed several genes for motility, toxins and extracellular enzymes which have been verified as the main bacterial virulence factors in several aquatic pathogenic bacterial species. Although the genome of the bacterium contained many important virulence genes, precise functional analysis is required to confirm this finding.

## Materials and Methods

### Comparative genome study

The bacterium *O.* sp. GK1 was deposited at the Iranian Biological Resource Center under the accession number of IBRC-M 10197. The complete genome sequence of *O.* sp. GK1 has been reported previously (1). For comparative genome analysis, three pathogen *Aeromonas* species (*Aeromonas hydrophila* subsp. *Hydrophila* ATCC 7966, *Aeromonas salmonocida* subsp. *salmonocida* A449 and *Aeromonas veronii* B565) were selected due to their close phylogenetic relationship with *O.* sp. GK1. Whole genome data of the three *Aeromonas* species was obtained from NCBI genomes. Comparative genomic study of *O.* sp. GK1 and the three closely related pathogenic *Aeromonas* species was done using MetaCyc database of metabolic pathways and enzymes (26). The Committee for Ethics in Iranian Biological Resource Center confirmed the study. 

### Phylogenetic analysis

Whole genome BLAST analysis was carried out using the Integrated Microbial Genome (IMG) system (27). Phylogenetic analysis of *O.* sp. GK1 was performed based on 16S rRNA gene sequence. Full length of 16S rRNA gene sequences were obtained from EzTaxon-e server (28) for nineteen members of *Aeromonadaceae*. The phylogenetic tree was constructed by neighbor-joining algorithm using MEGA5 software (29) with Escherichia coli KCTC-2441 being selected as an outgroup.

## Results

### Phylogenetic analysis

**Fig.1:**
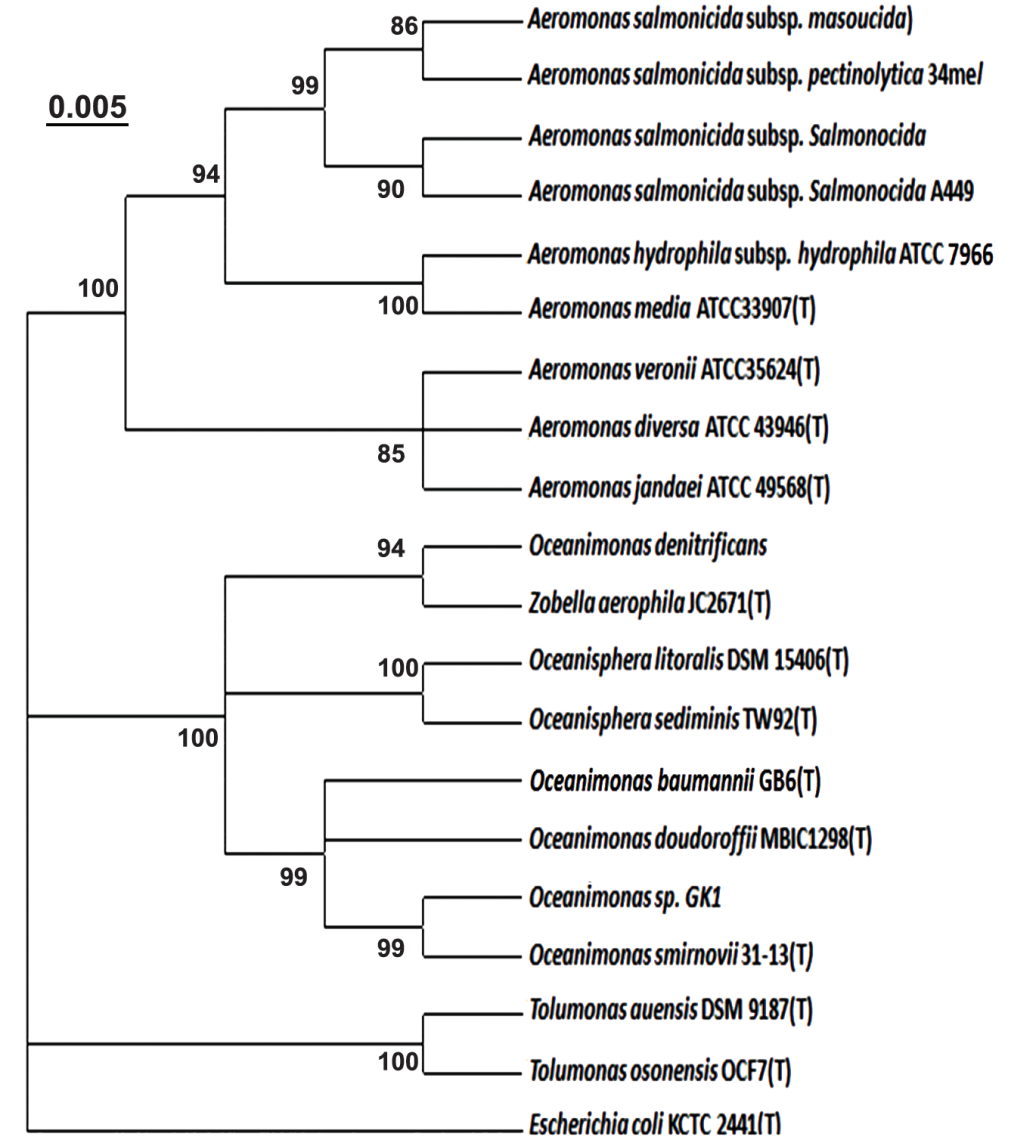
Phylogenetic tree based on 16S rRNA gene sequences constructed by neighbor-joining method showing the relationship between strain *Oceanimonas* sp. GK1 and its close relatives within members of the *Aeromonadaceae* family. Bootstrap percentages (based on 1000 replicates) of 70% are shown at branch points. Bar represents 0.005 substitutions per nucleotide position. Escherichia coli KCTC2441 was selected as an outgroup.

**Fig.2:**
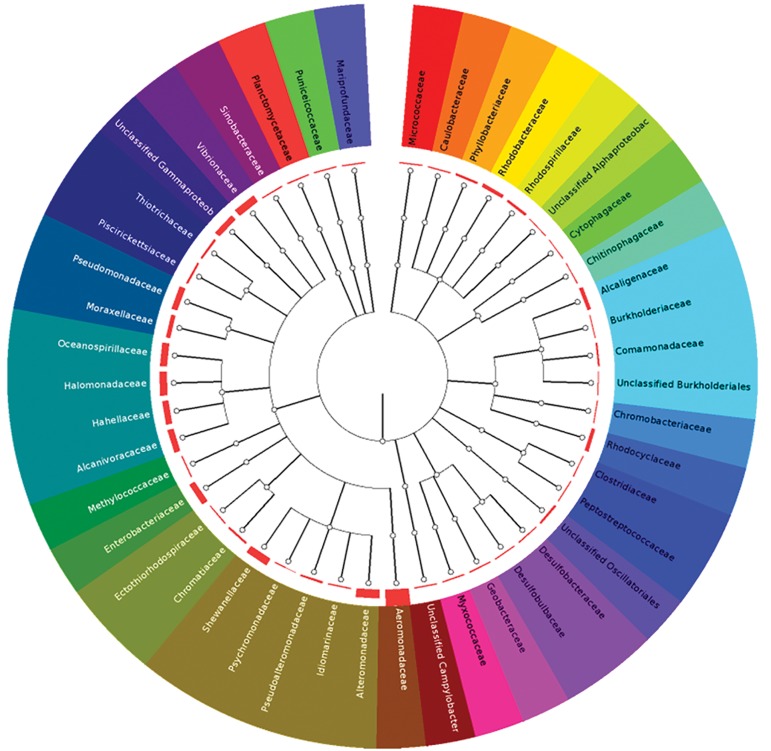
Genome scale BLAST analysis of the *Oceanimonas* sp. GK1 revealed the most similarity of the bacterium with the *Aeromonadaceae* family in contrast to other Gama proteobacteria. Also, the bacterium showed high similarity with Shewnellaceae and Entrobacteriaceae families in genome scale analysis. The figure was obtained from Integrated Microbial Genome (IMG) system.

Genome scale BLAST analysis revealed that *O.* sp. GK1 has the highest similarity with the *Aeromonadaceae* family in contrast to other Gamaproteobacteria. Also, this bacterium showed high similarity with the *Shewnellaceae* and *Entrobacteriaceae* families (Fig .1). Moreover, the phylogenetic tree derived from the neighbor-joining method based on the 16S rRNA gene sequences showed that *O.* sp. GK1 has the closest phylogenetic relationship with the other members of O. genus (Fig .2).

### Genome features

kThe *O.* sp. GK1 genome consists of a single circular chromosome of about 3.51 Mbp with 61.1 % GC content, and two plasmids with about 8.46 kbp and 4.24 kbp in length. In total, the genome codes for 3221 proteins and 112 structural RNAs. Several genes encode for choline/carnitine/betaine as well as proline/ glycine betaine transport systems, which are known as adaptive strategies of halophilic bacteria to salinity and thermal stresses (1, 30). Although plasmid annotation revealed neither antibiotic resistance nor virulence genes, the chromosome of *O.* sp. GK1 possesses several putative virulence genes (Table 1). Some of these genes are shared among all four genomes while some are unique to *O.* sp. GK1.

**Table 1:** List of virulence genes presents in Oceanimonas sp. GK1 genome


Virulence function	Gene name	Gene locus in Oceanimonas sp. GK1

Adhesion and biofilm formation	*Lateral flagella*	From GU3_13930 to 14120
	*Type IV pillin*	From GU3_14945 to 14965
		From GU3_ 15370 to 15385
		From GU3_ 04795 to 04815
	*ompAII*	GU3_12325
	*Murein lipoprotein*	GU3_11225
Enzymes	*Zinc metalloprotease*	GU3_06400
	*DegQ Serine protease*	GU3_04245
	*Membrane-bound serine protease (ClpP class)*	GU3_06290
	*Urease*	From GU3_08890 to 08920
	*Enolase*	GU3_15135
Toxins	*Zonular occludens toxin*	GU3_10305
	*Thermostable Hemolysin*	GU3_02870
	*RTX A*	GU3_12735
Antibiotic and drug resistance	*Multidrug efflux pumps and proteins*	GU3_02680, GU3_09445
		GU3_09470,GU3_09475
		GU3_10125, GU3_10715
		GU3_11340, GU3_13155
		GU3_13675, GU3_13795
		GU3_14635, GU3_14865
		GU3_15950,
	*Bicyclomycin resistance protein*	GU3_10430
Iron acquisition	*TonB-dependent siderophore receptor*	(GU3_ 15315)
	*TonB-dependent receptor*	(GU3_13825),(GU3_10775), (GU3_ 00520)


### Putative virulence factors

#### Adhesins

**Fig.3:**
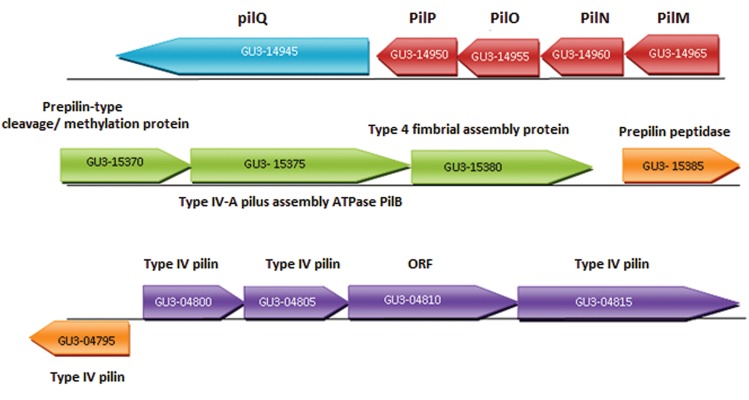
Operons containing genes coding for Type IV pilus in *Oceanimonas* sp. GK1 genome.

*In silico* analysis of the *O.* sp. GK1 genome revealed presence of complete sets of genes encoding polar and lateral flagella. Twenty nine genes code for the polar flagella system and eighteen genes for the lateral flagella system in *O.* sp. Gk1. Comparative genomic analysis of the two flagella systems of *O.* sp. GK1 with flagella systems of *Aeromonas* spp. revealed some differences between the genomes. In summary, in *A. salmonicida* subsp. *salmonicida* A449 genome, twenty two genes code for the components of polar flagella, and ten genes for the lateral flagella system. *A. hydrophila* subsp. hydrophila ATCC 7966 and *A. veronii* B565 genomes possess twenty one and twenty genes for polar flagella, respectively with no genes for lateral flagella in their genomes. Also, the *O.* sp. GK1 genome carries several operons containing type IV pillin genes (Fig .3). All the genes are shared in the four genomes compared with slight differences in operon arrangements. The orthologue for *O.* sp. GK1 prepilin-type cleavage/methylation protein encoding gene (GU3_15370) codes for TapA in *A. salmonicida* subsp. *salmonicida* A449. In *O.* sp. GK1, one gene (GU3_12325) was detected as OmpAII surface layer protein. For which its orthologues were found in *A. salmonocida* A.449 (ASA_1266), *A. hydrophila* ATCC 7966 (AHA_1280) and *A. veronii* B565 (B565_2931). Moreover, the orthologue gene for *Aeromonas* spp. major adhesion Aha1 was found in the *O.* sp. GK1 genome (GU3_11555). The other adhesin in *O.* sp. GK1 is murein lipoprotein encoding gene (GU3_11225). The gene is unique to *O.* sp. GK1 with no orthologue genes in the three *Aeromonas* species.

#### Secreted enzymes

The genome of *O.* sp. GK1 contains a zinc metalloprotease encoding gene (GU3_06400) and metalloprotease encoding genes (GU3_05090, GU3_12955). Orthologues were identified in the *Aeromonas* species genomes. Also, genome analysis revealed one gene as DegQ serine protease in the *O.* sp. GK1 (GU3_04245) and one gene for membrane-bound serine protease (ClpP class) (GU3_06290). The other important cytoplasmic enzyme which is encoded in the *O.* sp. GK1 genome is enolase (GU3_15135). 

Also, the *O.* sp. GK1 chromosome contains complete gene sets for urease subunits ( gamma (GU3_08895), beta (GU3_08900) and alpha (GU3_08905)) and urease accessory proteins (GU3_08910, GU3_08915, GU3_08920, GU3_08925) with nickle cation binding function. This enzyme and its related operons are unique to the *O.* sp. GK1. Furthermore, the chromosome carries thirty six genes and ORFs which code for transposases and phage integrases involved in mobile elements like insertion sequence (IS) elements and transposons. These genes are unique in the *O.* sp. GK1 genome. Nevertheless, several unique genes for transposases and integrases are present in *A. salmonocida* A449, and *A. veronii* B565, but no transposase encoding genes and IS elements could be found in *A. hydrophila* ATCC 7966. 

#### Toxins

Genome wide analysis revealed the presence of an encoding gene (GU3_10305) for Zonular Occludens Toxin (ZOT) in the *O.* sp. GK1 genome. The predicted protein was characterized as a protein with 358 aa in length and 41.838 KD molecular weight. The gene showed 41% homology with its orthologue in shewanella baltica. No orthologue of this gene was found in the three *Aeromonas* species genomes. The identified zot gene stands in Genomic Island 3 (GEI3), linked with a coding gene for type II and III secretion system protein (GU3_10300). GEI3 also contains genes for an integrase (GU3_10290) and phage replication initiation factor (GU3_10320). 

The *O.* sp. GK1 genome possesses an extracellular RTXA toxin gene (GU3_12735). This is present in the other three species of this study. 

Furthermore, one gene (GU3_02870) codes for a thermostable hemolysin. The predicted protein in *O.* sp. GK1 was characterized as a cytoplasmic protein with 214 aa in length and 23.917 kD molecular weight (based on nucleotide sequence). The orthologue genes in *A. hydrophila* ATCC 7966 (AHA_3217) and *A. veronii* B565 (B565_0938) as well as *Vibrio cholerae* and *Vibrio parahaemolyticus* code for the same product. Although, several other genes for extracellular cytotoxic hemolysins and extracellular earolysins exist in *A. hydrophila* ATCC 7966 (AHA_1512, AHA_0438) and in *A. salmonicida* A449 genomes (earA and earB) respectively, no orthologue genes of earolysins were found in *O.* sp. GK1. 

#### Iron acquisition

The genome of *O.* sp. GK1 includes an operon containing 3 genes for TonB-dependent receptor (GU3_13825), biopolymer transport exbB1 protein (GU3_13830) and tonB system transport protein ExbD1 (GU3_13835). Also tonB-dependent heme/hemoglobin receptor (GU3_02895), tonB-dependent siderophore receptor (GU3_ 15315), tonB-dependent receptor plug domain (GU3_00520) and tonB-dependent receptor (GU3_10775) were predicted as outer membrane proteins involved in iron acquisition. 

## Discussion

Specific adhesion of microorganisms to the animal or human host cell is the initial event in infectious diseases. Microbial adhesion is mediated by several types of adhesins such as flagella, pili and surface layer proteins. Flagella are surface structures which provide bacterial motility, however, it seems that they have more function than locomotion alone. Many studies have demonstrated the contribution of flagella to pathogenicity and virulence through chemotaxis, adhesion and invasion of host surfaces (31, 32). Some bacterial species such as *Aeromonas* spp. and *Vibrio parahaemolyticus* express two flagella systems (polar and lateral flagella) which are responsible for swimming in liquid and swarming motility (which allows bacteria to move over solid surfaces), respectively. Studies on lateral flagella have verified the role of this system in colonization, biofilm formation and bacterial virulence (33-37). The *O.* sp. GK1 genome contains several genes encoding polar and lateral fellagella which are shared among all four studied genomes with minor differences. Among the three studied pathogenic *Aeromonas* species, *Aeromonas salmonicida* subsp. *salmonicida* A449 has been previously characterized as a non-motile bacteria due to frameshift and indel mutations having occurred in genes related to both types of flagella (38). Fimbria or pili, a group of straight, ﬁlamentous structures on the bacterial surface (other than flagella) which are known as major bacterial adhesive structures, are composed of identical protein subunits called pilin and thought to be important virulence factors. Among the various types of pili, type IV pili have been well identified for their functions in adherence to host cell surfaces and virulence, twitching motility, modulation of target cell specificity and bacteriophage adsorption (39, 40). The role of type IV pili has been demonstrated in virulence of enteropathogenic *E. coli, Pseudomonas aeruginosa, Vibrio cholerae*, and some other pathogenic bacteria (41-43). According to the genome wide analysis results of *O.* sp. GK1, its genome possesses complete gene sets for type IV pili. The *O.* sp. GK1 prepilin-type cleavage/methylation protein coding gene is an orthologue of TapA in *A. salmonicida* A449. TapA has been shown to have a role in host invasion (38, 44). Among the bacterial surface proteins, members of the outer membrane protein A (OmpA) family as major outer membrane proteins of Gramnegative bacteria, are demonstrated to be important virulence factors (45). Important roles of OmpA protein in biofilm formation, adhesion and interaction of pathogenic bacteria with host cells have been verified in some pathogenic bacteria such as E.coli (45), *Acinetobacter baumannii* 19606 (46), *Aeromonas* veronii (47) and *Pasteurella multocida* (48). Aha1 adhesion protein is another outer membrane protein, known to be a key virulence factor of *A. hydrophila* in fish disease (49). Also, murein lipoprotein which is one of the major outer membrane components of infectious Gram-negative bacteria, contributes to bacterial pathogenicity (50). Mining the genome showed that genes coding for OmpAII, Aha1 and murein lipoprotein surface proteins exist in *O.* sp. GK1. In essence, *O.* sp. GK1, due to its lateral flagella system, type IV pili and surface adhesion proteins, may have the ability of colonization and biofilm formation which are the first steps of pathogenicity.

The other group of virulence factors is extracellular enzymes secreted by bacteria and fungi. Some of these secreted enzymes with virulence functions which have been verified for their key roles in infectious diseases include serine proteases (51), zinc metalloproteases (52), bacterial collagenases (53), chitinases (21), enolases (54), elastases (55) and phospholipases (20). The O. sp. GK1 genome carries several genes encoding DegQ and the clp-class of serine proteases, metalloproteases, enolase, urease and transposases. DegQ serine protease has been demonstrated to act as an important virulence factor in *Salmonella enterica* serovar typhimurium infecting mice. The essential role of the clp class of serine protease in intracellular parasitism and virulence of *Listeria monocytogenes*, has also been defined previously (56, 57). Enolase is an important enzyme which has received a lot of attention not only for its vital metabolic and biological roles, but also for its contribution to pathophysiological processes as well as bacterial disease and autoimmunity. Although enolase is a cytoplasmic enzyme, it can be found on the surface of certain eukaryotic cells (e.g., cancer, neuronal and some hematopoietic cells) and several pathogenic bacteria (e.g., *Streptococci* and *Pneumococci*) (54). When located extracellularly, the enzyme acts as a plasminogen receptor (58), contributing to pathogen-host interactions, bacterial colonization and bacterial migration into host cells. This enzyme has also been verified as a human plasminogen receptor in clinical *Aeromonas* hydrophila SSU (54). Urease is a nickle metalloenzyme and catalyzes the hydrolysis of urea to ammonia and carbamate providing a nitrogen source for the organism. A wide range of environmentally and medically important bacteria produce this enzyme. Most of the urease producing bacteria have the ability to differentially regulate the enzyme production based on the bacterial niche and environmental needs (59). In overt and opportunistic pathogenic bacteria, especially those inside the human body, the ability to activate this of the enzyme, when needed, is a critical factor for survival. The key role of the enzyme in pathogenicity of certain pathogenic bacteria has been confirmed previously (60-62).

According to our mined data, the genome of O. sp. GK1 contains genes encoding several important toxins such as ZOT, Repeats-in-toxin (RTXA), and thermostable hemolysin. ZOT is a novel toxin which was first reported in *Vibrio cholerae*. The toxin increases intestinal permeability by altering the structure of intercellular tight junctions (63). RTX is another toxin which has been reported as one of the most important virulence factors in pathogenic Gram negative bacteria such as *Vibrio cholerae* and *E. coli* (64-66) . Similarly, hemolysins are extracellular toxic proteins which are produced by many pathogenic Gram-negative and Gram-positive bacteria. Most hemolysins can lyse erythrocytes by forming pores of varying diameters in the membrane (67). Also, many of them are able to damage target mammalian cells almost certainly by a similar mechanism (68). Because of this cytolytic activity, the hemolysins are also named cytolysins and known as important virulence factors (69). Thermostable direct hemolysin of the marine bacterium "*Vibrio parahaemolyticus*" was reported as a virulence factor previously (70).

Iron acquisition is a key factor in biofilm formation and pathogenicity of some pathogenic bacteria. TonBdependent receptors which are present in the *O.* sp. GK1 genome are well studied for their critical function in iron uptake and virulence of pathogenic bacteria such as *Riemerella anatipestifer* (71), *Vibrio anguillarum* (72) and *Vibrio cholerae* (73).

Aquatic environments are natural habitats of many pathogenic bacteria in human and fish (74). The dynamic structure of the complex microbial communities in the niches with high rate of physicochemical changes provides the good conditions for bacterial-bacterial interaction and consequently increases the frequency of gene transfer. Finding the horizontal and vertical gene transfers may be more precise using genomic and metagenomics approaches. In the present study, based on the bioinformatics and information of various enriched databases, this is hypothesized that the non-pathogen *O.* sp. GK1 may changes to a hypothetical pathogen microorganism due to the evolutionary or genetically interactions with the pathogenic species of Vibrionaceae, Shewanellaceae and *Aeromonadaceae* in its niches.

## Conclusion

Although, conventional methods for detection of pathogenic bacteria are primarily based on cultivation procedures, detecting pathogens by means of target virulence gene amplification is considered as a sensitive method to be applied in environmental samples and food products. 

With the flourishing growth of Next Generation Sequencing (NGS) technologies, whole genome sequencing of the many clinically important bacteria has provided great deal of information, plenty enough to identify and characterize the virulence factors of a bacterium bypassing additional wet lab assays. 

Here, we show that one of the members of *Oceanimonas* genus contains putative virulence factors. The genome analysis of *O.* sp. GK1 represented several important virulence genes such as zot, rtx and hemolysins, serine proteases, enolase, urease, lateral flagella and type IV pili. Some of these are shared among the pathogenic species of *Aeromonads* and some are unique to *O.* sp. GK1. Although, we demonstrate putative pathogenicity of *O.* sp. GK1 at the genomic level, accurate functional characterization needs additional wet-lab studies. 
